# Cerebral hemodynamic monitoring of Parkinson’s disease patients with orthostatic intolerance during head-up tilt test

**DOI:** 10.1117/1.NPh.7.2.025002

**Published:** 2020-05-05

**Authors:** Jung Bin Kim, Zephaniah Phillips, Seung-ho Paik, Shin-young Kang, Nam-Joon Jeon, Byung-Jo Kim, Beop-Min Kim

**Affiliations:** a Korea University Anam Hospital, Department of Neurology, Seoul, Republic of Korea; b Korea University, Department of Bio-Convergence Engineering, Seoul, Republic of Korea; c Korea University Anam Hospital, Neurophysiology Laboratory, Seoul, Republic of Korea; d KLIEN Inc., Seoul Biohub, Seoul, Republic of Korea; e Korea University Anam Hospital, Brain Convergence Research Center, Seoul, Republic of Korea

**Keywords:** autonomic dysfunction, diffuse optical tomography, head-up tilt, orthostatic hypotension, Parkinson’s disease

## Abstract

**Significance:** Monitoring of cerebral perfusion rather than blood pressure changes during a head-up tilt test (HUTT) is proposed to understand the pathophysiological effect of orthostatic intolerance (OI), including orthostatic hypotension (OH), in Parkinson’s disease (PD) patients.

**Aim:** We aim to characterize and distinguish the cerebral perfusion response to a HUTT for healthy controls (HCs) and PD patients with OI symptoms.

**Approach:** Thirty-nine PD patients with OI symptoms [10 PD patients with OH (PD-OH) and 29 PD patients with normal HUTT results (PD-NOR)], along with seven HCs participated. A 108-channel diffuse optical tomography (DOT) system was used to reconstruct prefrontal oxyhemoglobin (HbO), deoxyhemoglobin (Hb), and total hemoglobin (HbT) changes during dynamic tilt (from supine to 70-deg tilt) and static tilt (remained tilted at 70 deg).

**Results:** HCs showed rapid recovery of cerebral perfusion in the early stages of static tilt. PD-OH patients showed decreasing HbO and HbT during dynamic tilt, continuing into the static tilt period. The rate of HbO change from dynamic tilt to static tilt is the distinguishing feature between HCs and PD-OH patients. Accordingly, PD-NOR patients were subgrouped based on positive-rate and negative-rate of HbO change. PD patients with a negative rate of HbO change were more likely to report severe OI symptoms in the COMPASS questionnaire.

**Conclusions:** Our findings showcase the usability of DOT for sensitive detection and quantification of autonomic dysfunction in PD patients with OI symptoms, even those with normal HUTT results.

## 1 Introduction

Orthostatic intolerance (OI) is a form of autonomic dysfunction in which symptoms manifest due to postural changes. OI can have many symptoms, ranging from orthostatic hypotension (OH) [i.e., a sustained drop in blood pressure (BP) due to orthostatic stress] to syncope.^1^ OH is the most common OI symptom for patients with Parkinson’s disease (PD), even in the early stages of the disease.^2^^–^^5^ In PD patients, OH is related to increased postural sway,^6^ risk of falling injury,^7^^,^^8^ cardiovascular events,^9^ and cognitive impairment.^10^ Therefore, OH can impair the quality of life and increase mortality rates among patients with PD. Considering the high prevalence and detrimental effects of OH in PD patients, timely detection and management of OH is critical to promote favorable functional outcomes and to decrease mortality rates.

The head-up tilt test (HUTT) is a widely used tool for the diagnosis of various OI symptoms. In short, the subject begins the examination laying on a table in supine position and then undergoes a dynamic tilt period (from supine to 70-deg tilt), a static tilt period (remained tilted at 70 deg), and a post tilt period (back to supine). Typically, the results of HUTT performance are based on serial monitoring of BP during dynamic and static tilting.^11^ HUTT performance can be used to diagnose an OI symptom if BP changes during orthostatic stress surpasses recommended clinical thresholds. All other HUTT performances are classified as a normal test result, implying that BP changes did not surpass the thresholds for OI symptoms.

The failure of the body’s autoregulatory system for maintaining cerebral perfusion in response to orthostatic challenges is thought to be responsible for the symptoms of OH.^12^ As BP is an indirect measurement of cerebral perfusion, quantifying BP changes during tilting by a conventional HUTT may not accurately reflect cerebral perfusion changes after orthostatic stress. In addition, diagnosis of OH in patients with PD using the HUTT might be limited due to the low reproducibility and sensitivity of the HUTT.^13^^,^^14^ Therefore, tools that can directly measure cerebral hemodynamic status are required for accurate diagnosis and assessment of OI symptoms, such as OH, in patients with PD.

In our previous studies, we have shown the feasibility of direct cerebral perfusion monitoring with near-infrared spectroscopy (NIRS) during the HUTT and Valsalva maneuver (VM).^1^^,^^15^ Specifically, patients with OH showed a delayed change in cerebral hemodynamics during the VM^15^ and a later inflection point of blood volume restoration during the HUTT compared with those of healthy controls (HCs),^1^ suggesting an impairment of the autonomic reflex, which maintains cerebral perfusion in patients with OH. These findings imply that direct monitoring of cerebral hemodynamics may provide additional information useful for the accurate diagnosis of OI symptoms.

Diffuse optical tomography (DOT) is an objective and validated method, which can resolve hemodynamic changes throughout the entire modeled medium using source–detector (SD) geometry,^16^ while NIRS is based on channelwise comparisons. Expanding upon our previous studies, we hypothesize that direct cerebral perfusion measurements using DOT may be a more sensitive tool for accurately characterizing cerebral hemodynamic patterns of PD patients with OI symptoms. Through hemodynamic monitoring, we aim to distinguish between PD patients with OH symptoms (PD-OH), PD patients with normal HUTT results (PD-NOR), and HCs. In addition, we aim to further investigate cerebral perfusion patterns of PD-NOR patients whose BP changes during the HUTT were within appropriate limits, yet the patients still suffer from OI symptoms.

## 2 Material and Methods

### 2.1 Subjects

Patients with PD who had symptoms suggesting OI were recruited for this study. Diagnosis of PD was based on the diagnostic criteria from the United Kingdom PD Society Brain Bank.^17^ We excluded patients with cognitive impairments and those who were unable to complete autonomic function tests (AFTs) and questionnaires without assistance. Patients were also excluded if they had cardiac arrhythmia or other medical conditions that could affect the results of AFTs. Motor function in PD was assessed using the Unified Parkinson’s Disease Rating Scale (UPDRS) Part III scores during the “off” period prior to AFTs.^18^ We applied the Hoehn and Yahr (H&Y) stage to categorize the global severity of PD.^19^ Cognitive function was measured by the mini-mental state exam (MMSE) and Montreal cognitive assessment (MoCA) at recruitment. Healthy volunteers without any OI symptoms and who did not have any medical condition that could affect the results of the AFTs were recruited for controls. Written informed consent was obtained from all enrolled patients. The use of human material in this study conforms to the principles outlined in the Declaration of Helsinki, and this study was reviewed and approved by the institutional review board.

### 2.2 Autonomic Symptom Questionnaire

To assess the severity of autonomic dysfunction, all participants were assessed with the Korean version of the COMPASS 31 questionnaire.^20^ The COMPASS 31 consists of 31 items that measures six different domains related to the following: 4 OI items, 3 vasomotor items, 4 secretomotor items, 12 gastrointestinal items, 3 bladder items, and 5 pupillomotor items. The total score of the COMPASS 31 ranges from 0 to 100, with higher scores indicating more severe autonomic dysfunction.

### 2.3 Autonomic Function Tests

All participants were requested to abstain from any medication, alcohol, or coffee that could affect autonomic function for at least 24 h before the test. Tests were performed in the following sequence according to the standard electrodiagnostic laboratory environment:^21^ (1) quantitative sudomotor axon reflex test, (2) heart rate response to deep breathing, (3) VM, and (4) HUTT. The composite autonomic severity scores (CASS), a validated measurement of the severity of autonomic dysfunction, was derived from the previously mentioned AFTs.^22^ Detailed methods of each AFT are described in our previous studies.^1^^,^^5^ Patients were classified as OH if a reduction of systolic BP of at least 20 mmHg or diastolic BP of at least 10 mmHg was seen within 3 min of standing up following the HUTT.^23^ Within our patient group, OH was the only observed OI symptom. All other patients were classified as PD-NOR. In the case that specific symptoms developed during the HUTT, patients were returned quickly to the supine position. Therefore, the static tilt time duration varied between subjects. For consistency, our time-series analysis was limited to 3 min of HUTT.

### 2.4 Diffuse Optical Tomography

**Fig. 1:**
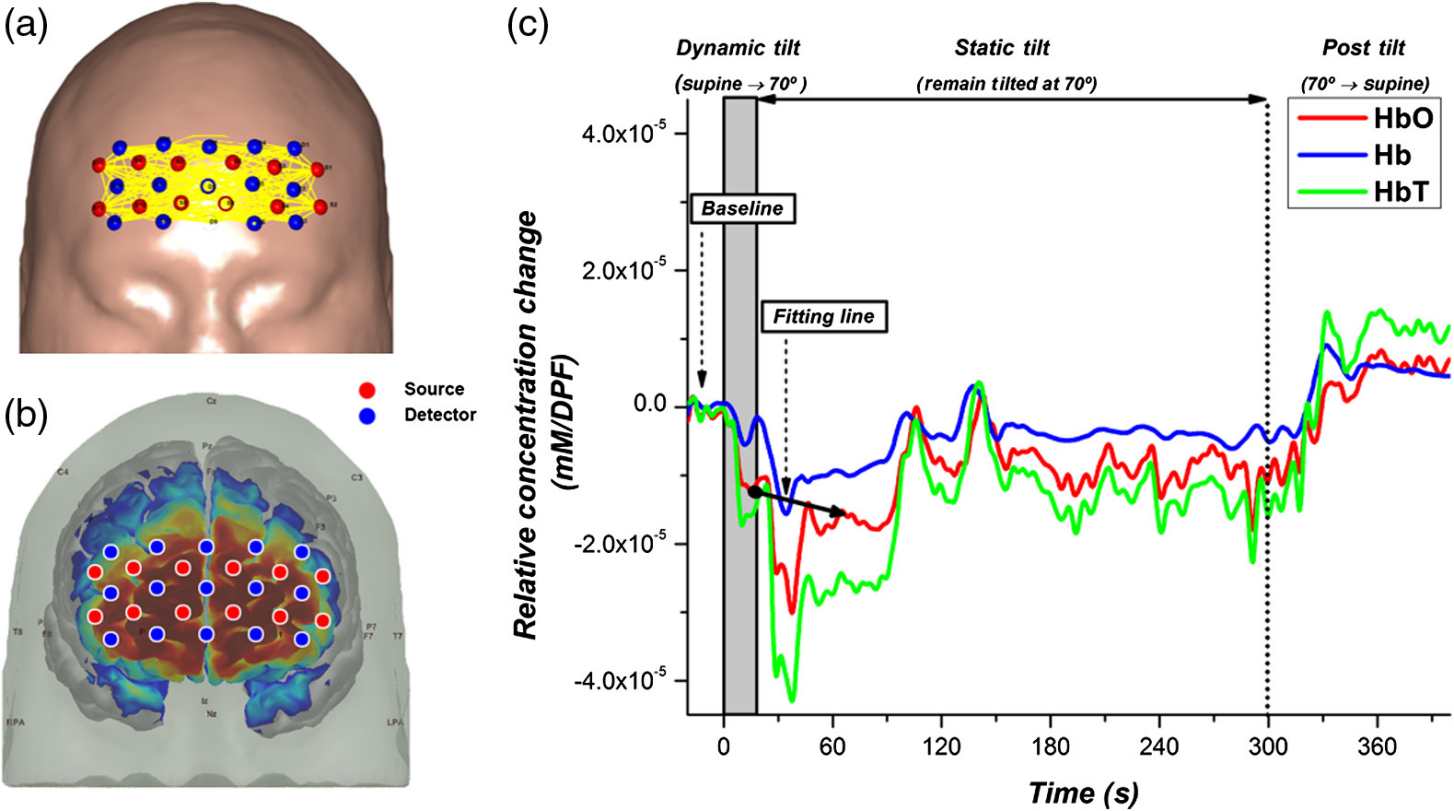
(a) Approximate placement of sources and detectors of the DOT system on the prefrontal area of the subject. (b) Sensitivity of DOT system, rendered in AtlasViewer. (c) Example of hemodynamic changes during the entire HUTT for one healthy control. The baseline period from which relative hemodynamics are calculated is indicated on the graph. In addition, an example of the fitting line used to quantify the rate of hemodynamic changes is indicated on the graph.

A continuous-wave 108-channel DOT system, comprising 15 detectors and 12 sources, was constructed and spanned the entire forehead. The system used in this study is the same as used in our previous studies.^1^^,^^15^ In short, the probe was controlled by an 8 bit of microcontroller unit with 200 to 4095 resolution. The system alternated LED sources between wavelengths of 760 and 830 nm at a temporal frequency of 5 Hz for scanning the entire forehead. The power emitted from the LED sources was ∼4  mW. Although the system had Bluetooth capability for transmitting data, it remained connected to a laptop in the room during the experiments. The bottom of the probe was aligned approximately along the Fp1-FpZ-Fp2 line and then secured to the subject with a band. The 108 channels were composed of 40 channels with an SD distance of 15 mm, 20 channels with an SD distance of 30 mm, 32 channels with an SD distance of 36 mm, and 16 channels with an SD distance of 45 mm. As shown in Fig. 1, the sensitivity profile of the probe spans the prefrontal area, based on the Colin27 MRI template.^24^ The data were collected and then processed offline in MATLAB 2013b (The MathWorks, Inc., Natick, Massachusetts).

Figure 1(c) shows an example of hemodynamic changes, averaged from the overall prefrontal area, during the entire HUTT for one HC subject. The figure indicates the baseline period from which relative hemodynamic changes are calculated and an example fitting line that is used to quantify the rate of hemodynamic changes. Each subject was instructed to avoid large motions that could induce motion artifacts in the signal. The data preprocessing steps were similar to our previous publications, including a 2-Hz low pass filter.^1^^,^^15^ In short, a wavelet-based denoising method (Daubechies 5) was applied to the time series data to remove large motion artifacts. This method has been shown to be effective in removing sudden and drastic changes in time series data induced by motion artifacts.^25^ Linear DOT calculations were performed with spatially variant regularization to increase the sensitivity in deeper regions of the brain.^26^^,^^27^

### 2.5 Statistical Analysis

Basic characteristics, H&Y stages, UPDRS part III, COMPASS, CASS, MMSE scores, MoCA scores, as well as relative changes in HbO, Hb, and total hemoglobin (HbT), were compared between the HC, PD-OH, and PD-NOR groups using an independent t-test and chi-square test, where appropriate (P<0.05). According to our analysis of blood perfusion patterns for the three groups, we subgrouped the PD-NOR group based on the rate of HbO change during the HUTT. Partial correlation analysis was performed between the rate of HbO change subgroupings during tilting and COMPASS scores, controlling for age (P<0.05).

## 3 Results

Forty-one PD patients with OI symptoms as well as seven HCs (age: 68.1±4.5, male: 3) were recruited for this study. Two patients were excluded from the final analysis because of low light intensity or large motion artifacts in their DOT data. Based on the HUTT findings, 10 patients were classified as PD-OH (age: 71.9±9.1, male: 5) and 29 as PD-NOR (age: 68.7±9.2, male: 20). Demographics and clinical data from the study participants are presented in Table 1. The PD-OH group and PD-NOR group did not differ in age, sex, proportion with hypertension, H&Y stage, UPDRS part III, MMSE, MoCA, or COMPASS scores. Metrics related to PD were not obtained for the HC group, since they did not show any symptoms of the disease. The PD-OH group had higher CASS than the PD-NOR group (4.6±1.9 versus 3.1±1.8, P=0.049).

**Table 1:** Demographics and clinical characteristics.

	PD-OH (n=10)	PD-NOR (n=29)	Healthy (n=7)	P (PD-OH versus PD-NOR)
Age, years	71.9±9.1	68.7±9.2	68.1±4.5	0.343
Male, n (%)^a^	5 (50.0)	20 (69.0)	3 (42.9)	0.446
Hypertension, n (%)^a^	4 (40.0)	14 (48.3)	0	0.726
H&Y stage	2.3±0.5	2.1±0.9	—	0.346
UPDRS part III	28.5±11.9	23.5±13.1	—	0.291
COMPASS	31.7±21.5	18.8±19.3	—	0.085
CASS	4.6±1.9	3.1±1.8	—	0.049
MMSE	25.1±5.1	26.0±4.3	—	0.577
MoCA	20.6±6.7	22.4±6.3	—	0.511
Rate of HbO change ($\times 10^{-4}\;\mathrm{mM}/\mathrm{DPF}$)	−5.6±6.7	0.8±12.1	7.0±8.1	0.613

Note: Each value represents the mean value ± standard deviation. Independent t-test was used to compare the variables between the groups.

CASS, composite autonomic severity score; COMPASS, composite autonomic symptom score; H&Y, Hoehn and Yahr; HUTT, head-up tilt test; MMSE, mini-mental state exam; MoCA, Montreal cognitive assessment; OH, orthostatic hypotension; PD, Parkinson’s disease; PD-OH, PD with OH; PD-NOR, PD with normal HUTT; UPDRS, unified Parkinson’s disease rating scale.

a Chi-square test was performed.

**Fig. 2:**
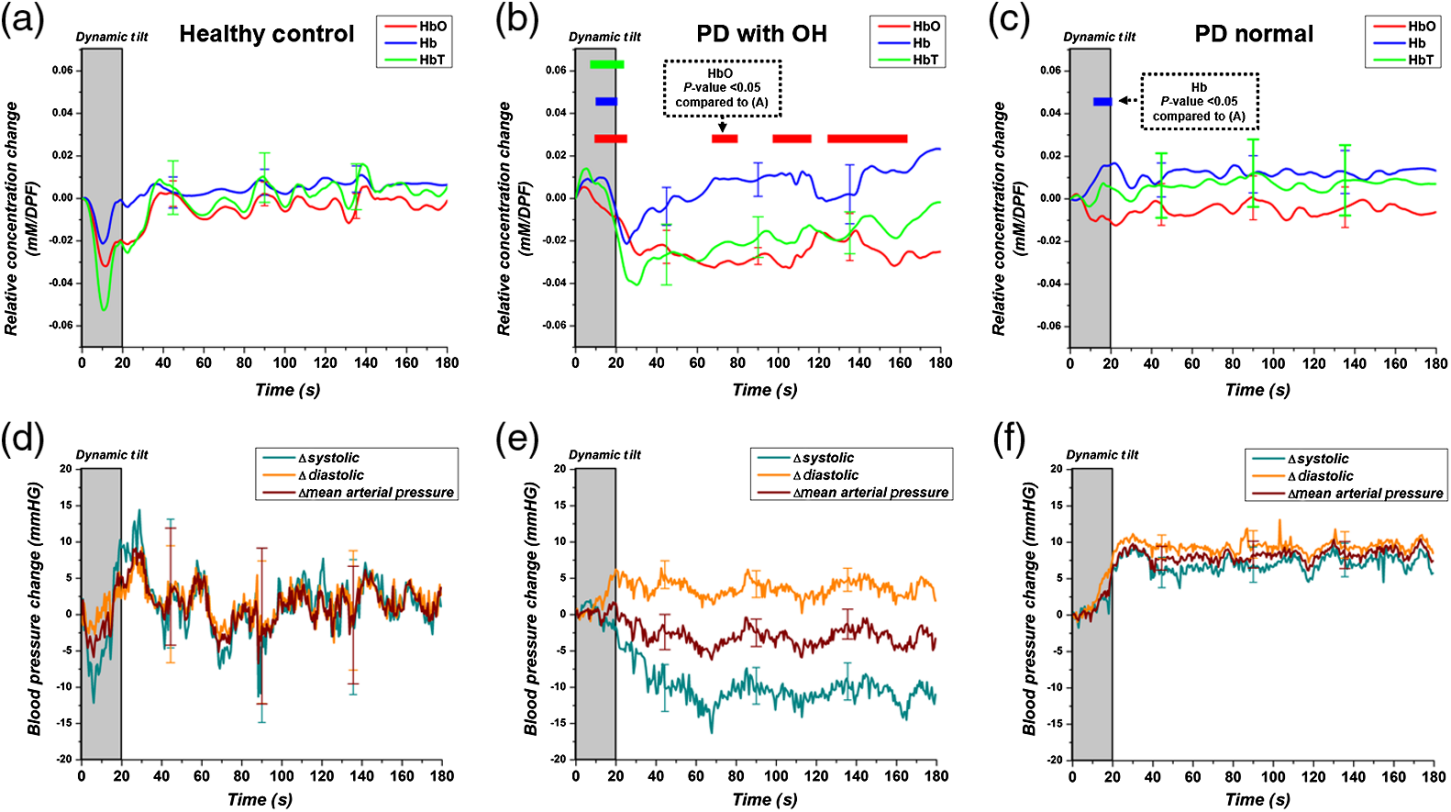
HUTT hemodynamic time series changes for (a) healthy controls, (b) PD with OH patients, and (c) PD normal. Hemodynamics changes include HbO (red), Hb (blue), and HbT (green). The gray-shaded area marks the period of dynamic tilting. A statistical difference (P<0.05) between PD groups and healthy controls is denoted with a colored bar over the time points. The color of the bar responds to the hemodynamic value that tested significantly different. (d)–(f) Average BP changes relative to start of HUTT from the (a)–(c) subject groups: changes in systolic pressure (green), diastolic (orange), and MAP (maroon).

Figure 2 shows the group averaged HbO, Hb, and HbT changes over the entire prefrontal area during the HUTT for HC, PD-OH, and PD-NOR groups. For HC, HbO and HbT drop during the dynamic tilt period and then overshoot in the early period of static tilt. For PD-OH, after a slight increase at the start of dynamic tilt, HbO and HbT continually decrease through the dynamic tilt period and into the static tilt. In contrast to the other groups, the average HbT response of the PD-NOR group did not change from the baseline throughout the dynamic and static tilt periods. This is due to the averaging effect of individual increasing and decreasing HbT changes within the PD-NOR group.

At each time point, a two-tailed t-test was performed between HCs and each PD patient group. Statistically significant time points (P<0.05) are marked in Fig. 2 with a bar over the time points, where the bar color corresponds to the hemodynamic change (e.g., statistically significant difference in HbT between the HC and PD-OH or PD-NOR groups is denoted with a green bar). Compared with the HC group, the PD-OH group showed a statistically significant increase in HbO, Hb, and HbT during the dynamic tilt period and a fall in HbO for the majority of the static tilt period. Although PD-NOR patients showed a large drop in HbO during dynamic tilt, this was because of a large outlier; therefore HbO did not test significantly different from HC. The rest of the time series did not test significantly different between PD-NOR and HC.

Average time series BP changes for subject groups during the dynamic tilt and static tilt periods are shown in Figs. 2(d)–2(f). BP changes were calculated relative to the start of the HUTT. As expected, HCs showed a recovery of BP during static tilt, after a fall during dynamic tilt, similar to the hemodynamic changes. PD-OH patients showed a continuous fall of systolic and mean arterial pressure (MAP), also similar to the hemodynamic changes. PD-NOR patients did not show a large drop of BP during dynamic tilt and lack the continuous fall of BP changes shown in the PD-OH group.

**Fig. 3:**
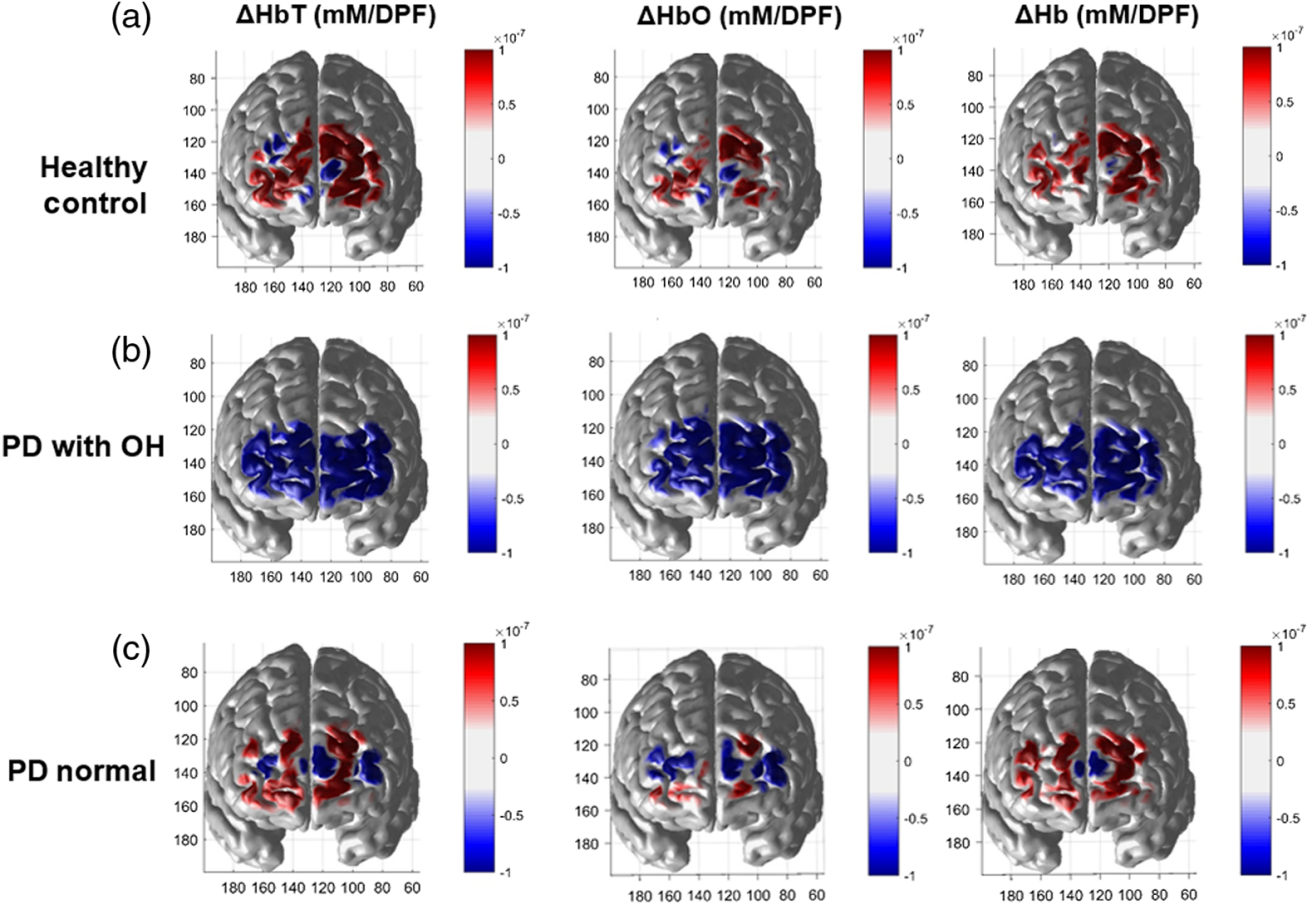
Group average of the difference in HbT, HbO, and Hb for (a) healthy controls, (b) PD patients with OH, and (c) PD patients with normal HUTT. A 5-s averaged DOT image at 30 to 35 s (∼10  s after start of static tilt) was subtracted from a 5-s averaged DOT image at 10 to 15 s within dynamic tilt.

DOT images depicting hemodynamic recovery during the static and dynamic tilt periods are presented in Fig. 3. A mean DOT image from 30 to 35 s (∼10  s after the start of static tilt) was subtracted from a mean DOT image at 10 to 15 s within the dynamic tilt period. As expected, the HC group showed a large increase in HbO, Hb, and HbT when comparing static tilt to dynamic tilt hemodynamics. In contrast, the PD-OH group showed a continuous decrease of HbO, Hb, and HbT within the early static tilt period. While the hemodynamic changes in the HC group were distributed mainly within the range of positive values, those in PD-NOR group showed a mixture of increasing and decreasing hemodynamic changes across the prefrontal area, suggesting that hemodynamic changes within this group are more diverse than within the other groups.

**Fig. 4:**
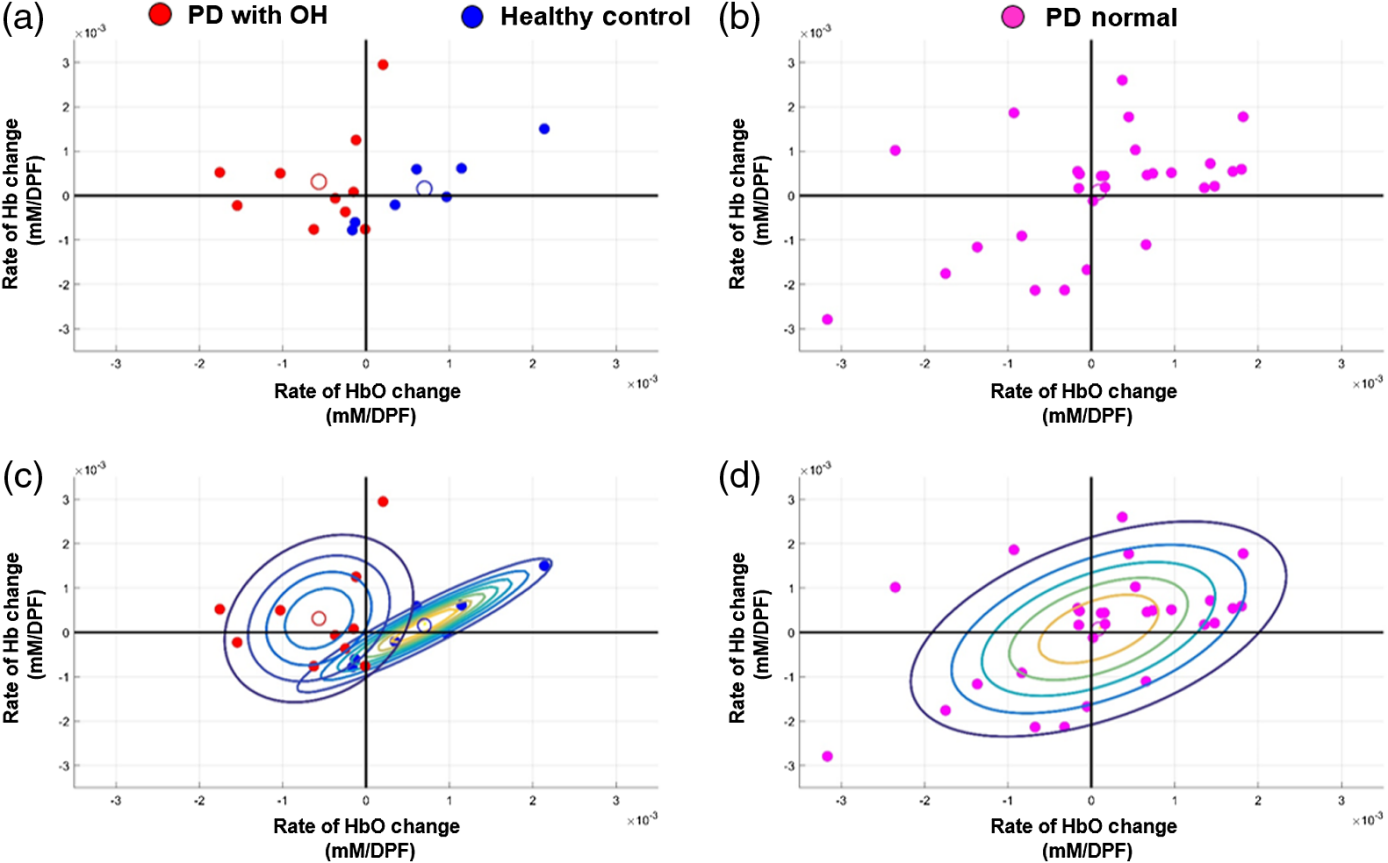
Rate of change for the linear fit of HbO (x axis) and Hb (y axis) from 15 to 60 s of tilting for individual healthy controls [blue circle in (a), (c)], PD with OH patients [red circle in (a), (c)], and PD patients with normal HUTT results [magenta circle in (b), (d)]. A Gaussian mixed model was fitted to each group and the centroid (outlined circle) of each group was determined through k-means clustering.

The rate of change for a linear fit of HbO and Hb values was calculated to quantify the hemodynamic recovery from the late dynamic tilt period to the early static tilt period [i.e., 15 to 60 s as shown in Fig. 1(c)]. The rate of change in HbO compared with the rate of change in Hb was plotted for individual subjects of the PD-OH and HC groups [Fig. 4(a) and separately for the PD-NOR group [Fig. 4(b)]. A Gaussian mixture model was fitted to each group to visualize the distinguishing features between groups. The centroid of each group, as calculated through k-means clustering, is marked with an outlined circle [Figs. 4(c) and 4(d)]. As depicted in the figure, the HC group tended to have a positive rate of HbO change, while the PD-OH group tended to has a negative rate in HbO change during the HUTT. The PD-NOR group had a widespread rate of HbO change, indicating that the group may consist of PD patients whose perfusion patterns resemble HCs and those who resemble the PD-OH group. Based on the rate of change in HbO, we subgrouped the PD-NOR group into two separate groups: PD-NOR with a positive rate of HbO change (n=17) and PD-NOR with a negative rate of HbO change (n=12). The rate of HbO change tested significantly different between the two PD-NOR subgroups (Table 2). However, the subgroups did not show any differences in age, sex, proportion with hypertension, H&Y stage, UPDRS part III, MMSE, MoCA, or CASS scores (Table 2).

**Table 2:** Demographics and clinical characteristics of Parkinson’s disease patients with normal HUTT results.

	Negative HbO (n=12)	Positive HbO (n=17)	P
Age, years	65.7±12.7	70.1±6.2	0.158
Male, n (%)^a^	7 (58.3)	13 (76.5)	0.422
Hypertension, n (%)^a^	4 (33.3)	10 (58.8)	0.264
H&Y stage	1.9±0.9	2.2±0.9	0.284
UPDRS part III	24.1±17.2	23.1±9.8	0.839
COMPASS	29.0±26.3	11.6±6.6	0.045
CASS	2.4±1.9	3.6±1.6	0.093
MMSE	25.5±5.7	26.3±3.3	0.636
MoCA	21.6±8.9	22.8±4.6	0.645
Rate of HbO change ($\times 10^{-4}\;\mathrm{mM}/\mathrm{DPF}$)	−9.9±9.9	8.5±6.3	<0.001

Note: Each value represents the mean value ± standard deviation. Independent t-test was used to compare the variables between the groups.

CASS, composite autonomic severity score; COMPASS, composite autonomic symptom score; H&Y, Hoehn & Yahr; HbO, oxyhemoglobin; MMSE, mini-mental state exam; MoCA, Montreal cognitive assessment; OH, orthostatic hypotension; PD, Parkinson’s disease; UPDRS, unified Parkinson’s disease rating scale.

a Chi-square test was performed. Negative HbO represents a negative rate of HbO change during tilting, and positive HbO represents a positive rate of the change.

The same linear fitting procedure to derive the rate of HbO change was performed for MAP, from 15 to 60 s of the HUTT. The average rate of MAP change is shown in Table 3. The $R^{2}$ was calculated from a linear fit between the rate of HbO change and the rate of MAP change. The low $R^{2}$ values indicate that the calculated rate of HbO change and rate of MAP change do not correlate well for any of the subject groups. These findings further demonstrate the new information that directs cerebral hemodynamic monitoring can provide over BP monitoring alone.

**Table 3:** Correlation of rate of HbO change and rate of MAP change.

	PD-OH	PD-NOR negative	PD-NOR positive	Healthy
Rate of HbO change ($\times 10^{-4}\;\mathrm{mM}/\mathrm{DPF}$)	−5.6±6.7	−9.9±9.9	8.5±6.3	7.0±8.1
Rate of MAP change ($\times 10^{-1}\;\mathrm{mmHG}$)	−0.9±1.5	0.2±1.4	0.6±1.7	−0.7±3.2
$R^{2}$	0.168	0.074	0.001	0.299

As mentioned previously, the COMPASS score quantifies the self-reported autonomic dysfunction symptoms for patients. Table 4 summarizes the distribution of COMPASS scores between the three PD patient groups. The positive rate of HbO change showed a significant difference in COMPASS scores compared with PD-OH patients. However, the negative rate of HbO change scores did not differ from the OH patents (Table 4). When comparing the two PD-NOR subgroups, the negative rate of HbO change had a statistically significant higher COMPASS scores than the positive rate of HbO change (P=0.04, not shown on the table). The rate of HbO change for the two PD-NOR subgroups negatively correlated with COMPASS (r=−0.324, P=0.047), controlling for age.

**Table 4:** COMPASS scores of PD-NOR subgroups and PD-OH.

	Mean	Median	P (compared to PD-OH)	
PD-NOR positive rate of HbO change	11±6.4	11	0.016	
PD-NOR negative rate of HbO change	29±25.1	18	0.79	
PD-OH	31.7±20.4	26	—	

**Fig. 5:**
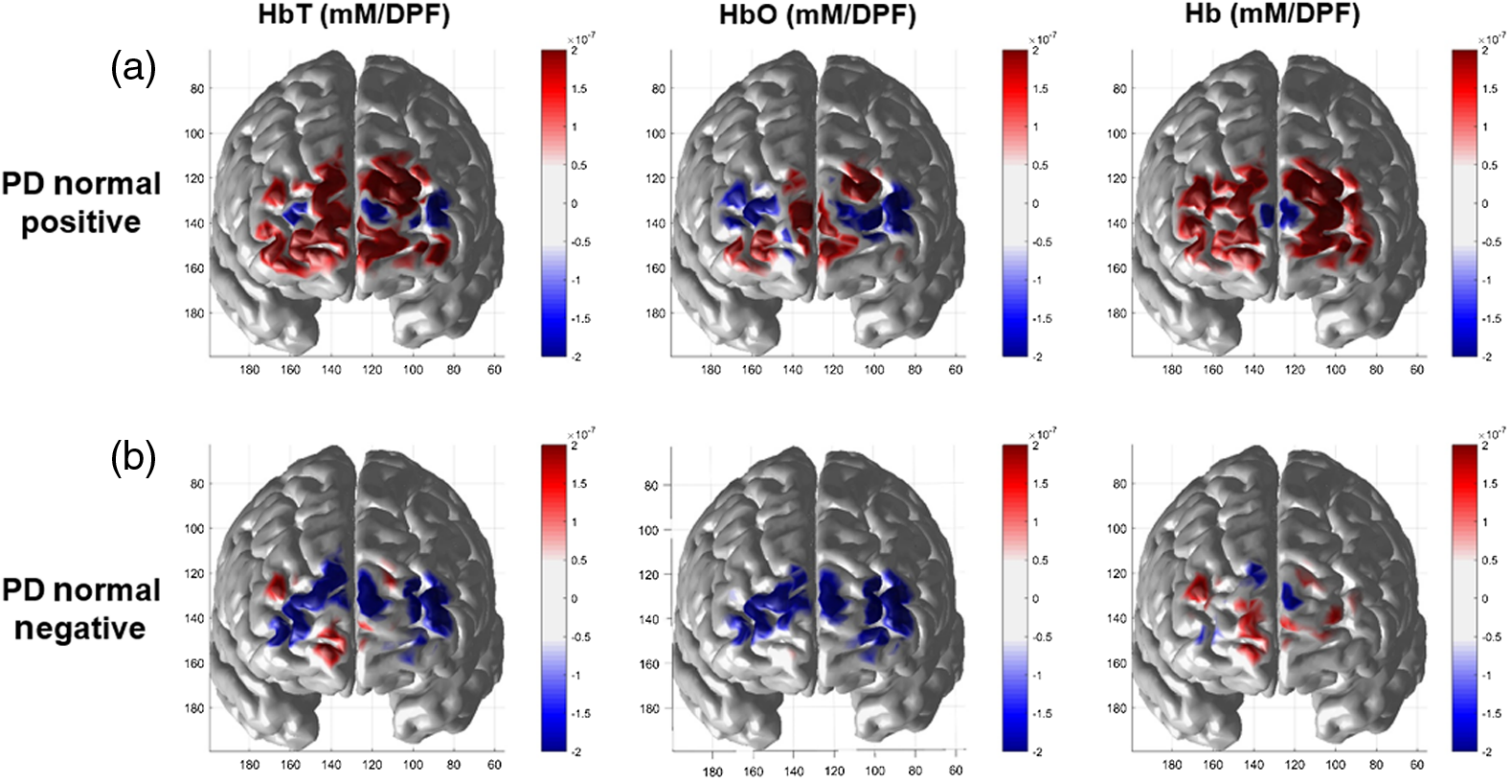
Group average of HbT, HbO, and Hb changes after 100 s of remaining in static tilt, for (a) PD normal with a positive rate of HbO change and (b) PD normal with a negative rate of HbO change. The DOT images were averaged over 10 s.

The group averaged cerebral hemodynamic changes for the two subgroups of PD-NOR after 100 s of remaining in static tilting is shown in Fig. 5. A 10-s average of HbT, HbO, and Hb was calculated for (a) PD-NOR with positive rate of HbO change and (b) PD-NOR with negative rate of HbO change. After remaining in static tilt, PD-NOR positive patients showed a large increase of HbT across the entire prefrontal area. Although PD-NOR positive patients do not show uniform increase of HbO across the prefrontal area, Hb increases uniformly. PD-NOR negative patients showed a large decrease in HbT. The drop of HbO for PD-NOR negative patients is consistent across the prefrontal area without a large change of Hb. The PD-NOR negative patient’s perfusion patterns closely resemble PD-OH’s trend of decreasing HbO and HbT, as shown in Fig. 2.

## 4 Discussion and Conclusion

DOT can be a useful method for monitoring cerebral oxygenation changes during the HUTT as it enables the quantification of hemodynamics changes including HbO, Hb, and HbT.^28^^–^^31^ Since the requirement for neuronal activity is oxygen metabolism, changes in HbO could reflect the oxygen demand of neuronal tissue.^32^^–^^34^ There is about 500 to 1000 ml of blood that pools toward the lower part of the body when standing up, which reduces cerebral blood flow and ultimately increases the demand for oxygen in brain tissue.^35^ In response to these hemodynamic changes, sympathetic outflow is activated to maintain cerebral perfusion during upright posture.^35^ Therefore, the rate of HbO changes during tilting might be considered a quantitative measurement of autonomic compensatory function in response to orthostatic stress.

Similar to our previous work, the HC group showed a rapid recovery in cerebral perfusion (HbO and HbT) after static tilt, whereas PD patients with OH showed a continual decrease in HbO and HbT.^1^ HCs and PD-OH patients can be distinguished by their positive and negative rates of HbO change during the HUTT, respectively. For the PD-NOR group, cerebral perfusion patterns were mixed. When subgrouped according to the rate of HbO change, patients with a negative rate of HbO change did not show a recovery in cerebral perfusion while those with a positive rate of HbO change showed a return of blood volume. PD-NOR patients with a negative rate in HbO had significantly higher COMPASS scores, demonstrating that their self-reported autonomic dysfunction symptoms are more aligned with their hemodynamic changes rather than their BP changes during the HUTT.

Given the above-mentioned mechanisms, we speculate that the negative rate of HbO change found in the PD-OH group and some PD-NOR patients suggests impaired autonomic function that may activate sympathetic vascular control in response to tilting. Similarly, a positive rate of HbO change in the HC group may be caused by a compensatory increase in cerebral blood flow. Our finding of an inverse relationship between the COMPASS questionnaire score and the rate of HbO change during tilting can support this speculation. Specifically, a higher rate of HbO change, suggesting a preserved compensatory autonomic function, was related to milder autonomic dysfunction symptoms as reported in COMPASS. In contrast, the lower rate of HbO change (toward negative values) reflects a failure in compensation and was related to more severe COMPASS-related autonomic dysfunction symptoms.

The quantitative discrimination between patients with normal or impaired autonomic function within the PD-NOR group suggests that DOT monitoring can complement the low sensitivity and poor reproducibility of the HUTT.^36^ Specifically, our results of PD-NOR suggest that DOT measurement may sensitively provide information that cannot be gleaned from conventional BP monitoring during the HUTT. Based on our findings, DOT could be applied to differentiate PD patients having OI symptoms as an alternative to HUTT with BP monitoring. Long-term observations of the progression of PD and OH in the two PD-NOR subgroups could be used to further support our findings.

Several limitations of this study should be noted. First, we averaged hemodynamic data from the overall prefrontal area to generally characterize the hemodynamic trends in the subjects. Since there is a degree of arbitrariness in determining the boundary of regions of interest for interpreting the clinical implications, we could not perform further regional analysis. Therefore, we could not discriminate between regions in the prefrontal area. Given the lateralization of autonomic control and the anatomical correlate for autonomic reflexes,^37^^,^^38^ further regional analysis is required to localize the autonomic hemodynamic responses during tilting. The ability of DOT to reconstruct hemodynamic changes across the prefrontal area (as shown in Figs. 3 and 5) may reveal additional clinical markers for PD patients. Moreover, during dynamic tilt, both PD groups showed an increase of either HbO and HbT [Fig. 2(b)] or just Hb [Fig. 2(c)]. However, the exact reason for this requires further investigation. Second, our study did not include OH patients without PD as a basis of comparison to understand the effect of PD on cerebral hemodynamic changes during the HUTT. Therefore, our results cannot be necessarily interpreted as PD-specific findings.

To the best of our knowledge, our study is the first application of DOT to PD patients with OI symptoms during the HUTT to explore the patterns of cerebral hemodynamic changes. Our findings provide additional insight into the utility of DOT for the sensitive detection and quantification of autonomic dysfunction in PD patients with OI symptoms, even in those with normal HUTT results. Further studies comparing follow-up HUTT findings between positive and negative rates of change in HbO during the HUTT are required to specify the role of DOT as a sensitive tool for predicting prognosis.
